# Choice between reinforcer delays versus choice between reinforcer magnitudes: Differential Fos expression in the orbital prefrontal cortex and nucleus accumbens core

**DOI:** 10.1016/j.bbr.2010.05.014

**Published:** 2010-12-01

**Authors:** S. da Costa Araújo, S. Body, L. Valencia Torres, C.M. Olarte Sanchez, V.K. Bak, J.F.W. Deakin, I.M. Anderson, C.M. Bradshaw, E. Szabadi

**Affiliations:** a Psychopharmacology Section, Division of Psychiatry, University of Nottingham, Room B109, Medical School, Queen's Medical Centre, Nottingham NG7 2UH, UK; b Neuroscience & Psychiatry Unit, Division of Psychiatry & Behavioural Sciences, University of Manchester, Stopford Building, Oxford Road, Manchester M13 9PT, UK

**Keywords:** Nucleus accumbens core, Orbital prefrontal cortex, Fos expression, Inter-temporal choice, Adjusting-delay schedule, Adjusting-magnitude schedule, Delay discounting, Rat

## Abstract

Lesions of the orbital prefrontal cortex (OPFC) and the nucleus accumbens core (AcbC) can disrupt performance in inter-temporal choice tasks, possibly by increasing the organism's sensitivity to delay and/or magnitude of reinforcement. This experiment examined whether exposure to an inter-temporal choice would induce neuronal activation in these areas, as indicated by enhanced expression of the Fos protein. Twelve rats were trained to press levers A and B under an adjusting-delay schedule in which a response on A delivered 50 μl of a sucrose reinforcer after 2 or 18 s, whereas a response on B delivered the same reinforcer after a delay that was adjusted in accordance with the rat's choices. Another 12 rats were trained under a similar schedule in which a response on A delivered an immediate reinforcer of size 20 or 180 μl, whereas a response on B delivered an immediate reinforcer whose size was adjusted in accordance with the rat's choices. A third group received training under a schedule that did not entail variation of reinforcer size or delay, or choice between reinforcers, and a control group underwent food restriction without behavioural training. Exposure to the adjusting-delay schedule was associated with enhanced Fos expression in both the OPFC and AcbC, whereas exposure to the adjusting-magnitude schedule was associated with enhanced Fos expression in the OPFC but not the AcbC, compared to the control group. The results are consistent with previous findings that implicated the AcbC and OPFC in delay discounting, and the OPFC in sensitivity to reinforcer size.

## 1 Introduction

In an inter-temporal choice schedule, the subject chooses between reinforcers that differ with respect to their sizes and delays. For example, a subject may be faced with a choice between a small reinforcer that is delivered soon after an operant response and a larger reinforcer whose delivery is considerably delayed. One important determinant of inter-temporal choice is the organism's sensitivity to delay of reinforcement, expressed by the hypothetical process of delay discounting whereby reinforcer value is postulated to be degraded as a function of the length of the response–reinforcer interval [2,3,24,27,34]. Another determinant of inter-temporal choice is the organism's sensitivity to different sizes of reinforcers, a principle that is recognized by various behavioural models of inter-temporal choice [14,29], and which is expressed by the economic principle of diminishing marginal utility [18,35,40].

The neural substrate of inter-temporal choice is incompletely understood, although considerable advances have been made in recent years [6,12,21,42]. Two structures that have been implicated in the regulation of inter-temporal choice are the core of the nucleus accumbens (AcbC) and the orbital prefrontal cortex (OPFC). Lesions of both these structures have been found to disrupt inter-temporal choice in animals [4,5,8,11,16,17,30,33,38,43]. The aim of the present experiment was to complement these findings by examining whether, in intact rats, performance of an inter-temporal choice task would give rise to detectable changes in neuronal activation in the AcbC and OPFC, as indicated by the levels of expression of Fos, the protein product of the immediate-early proto-oncogene *c-fos* [15,39].

A major difficulty encountered in the interpretation of experimental findings in this area arises from the complex nature of conventional inter-temporal choice schedules. Since these schedules generally entail choice between reinforcers that differ with respect to both size and delay, it is often difficult to establish whether the disruptive effect of an intervention has been caused by a change in the organism's sensitivity to one or other, or both, of these features of the reinforcers [13,14,20,29]. Mathematical models of inter-temporal choice offer one approach to disentangling the roles of delay discounting and sensitivity to reinforcer size [14,28]. For example, according to the ‘multiplicative hyperbolic model’ of inter-temporal choice [14], an extension of Mazur's [24,27] hyperbolic model of delay discounting, an organism's sensitivities to delay and size of reinforcement are represented by the parameters of separate hyperbolic functions, the overall value of each reinforcer in an inter-temporal choice task being computed by the multiplicative combination of the two functions. The model generates a null equation in which the indifference delay to the larger reinforcer (i.e. the delay to the larger reinforcer corresponding to equal choice of the two reinforcers) is linearly related to the delay to the smaller one. The effects of neurobiological interventions on this linear function may be used as a basis for inferring changes in the organism's sensitivity to reinforcer size and delay [14]. Using this methodology, evidence has been obtained that suggests that lesions of the AcbC may alter the rate of delay discounting without affecting sensitivity to reinforcer size [4], whereas lesions of the OPFC may alter the organism's sensitivity to both size and delay of reinforcement [16].

It should be noted that experiments employing mathematical analyses of inter-temporal choice generally do not dissociate the influences of delay and size of reinforcement experimentally. Conventional inter-temporal choice tasks are used which entail choice between reinforcers that differ with respect to both size and delay, and post-experimental mathematical analysis is employed to separate the influences of delay discounting and size sensitivity [14]. While this approach provides a means of assessing the effects of neurobiological interventions on delay discounting and size sensitivity, it is not suitable for assessing the engagement of different brain structures in these two processes in intact animals. What is needed is a behavioural schedule that entails choice between reinforcers that differ with respect to either size or delay, but not to both of these features. Such a schedule has been devised by Mazur [22].

In Mazur's [22] schedule, the subject makes repeated choices between two reinforcers of equal magnitude. Reinforcer A is delivered after one or other of two fixed delays, *d*_A(short)_ and *d*_A(long)_, which occur with equal probability (*p*[*d*_A(short)_] = *p*[*d*_A(long)_] = 0.5), whereas reinforcer B is delivered after a variable delay, *d*_B_, which is adjusted in response to the subject's choices in successive blocks of trials. If the subject displays a preference for A in trial block *n*, *d*_B_ is reduced in block *n* + 1; if B is preferred in block *n*, *d*_B_ is increased in block *n* + 1. The principal dependent variable in this schedule is the value of *d*_B_, which oscillates in response to the subject's choices, eventually approaching a quasi-stable value (the ‘indifference delay’, *d*_B(50)_), which lies between *d*_A(short)_ and *d*_A(long)_. The value of *d*_B(50)_, which is generally substantially shorter than the arithmetic mean of *d*_A(short)_ and *d*_A(long)_, is of considerable theoretical importance (see Section 4). However, the utility of the schedule for the present experiment lies in the fact that, unlike conventional inter-temporal choice tasks, it enables choice based on different delays to reinforcement to be examined in the absence of any confounding influence of different reinforcer sizes.

In the present experiment, an adjusting-magnitude schedule, analogous to the adjusting-delay schedule described above, was developed in order to assess choice based on reinforcer size uncontaminated by the effect of reinforcer delay. Reinforcer A comprised either a small or a large volume of a sucrose solution, whereas the size of reinforcer B was adjusted in successive blocks of trials in response to the subject's choices in the preceding block. Based on the evidence outlined above, it was predicted that exposure to the adjusting-delay schedule would induce neuronal activation in both the AcbC and the OPFC, whereas exposure to the adjusting-magnitude schedule would result in activation of the OPFC but not the AcbC.

## 2 Methods

The experiment was carried out in accordance with UK Home Office regulations governing experiments on living animals.

### 2.1 Subjects

Forty-four experimentally naive female Wistar rats (Charles River, UK) approximately 4 months old and weighing 250–300 g at the start of the experiment were used. They were housed individually under a constant cycle of 12 h light and 12 h darkness (light on 06:00–18:00 h), and were maintained at 80% of their initial free-feeding body weights throughout the experiment by providing a limited amount of standard rodent diet after each experimental session. Tap water was freely available in the home cages.

### 2.2 Apparatus

The rats were trained in standard operant conditioning chambers (CeNeS Ltd, Cambridge, UK) of internal dimensions 25 cm × 25 cm × 22 cm. One wall of the chamber contained a recess into which a peristaltic pump could deliver a 0.6 M sucrose solution. Two apertures were situated 5 cm above and 2.5 cm to either side of the recess, through which motor-operated retractable levers could be inserted into the chamber. The levers could be depressed by a force of approximately 0.2 N. A 2.8-W lamp was mounted 2.5 cm above each lever; a third lamp was mounted 10 cm above the central recess. The operant chamber was enclosed in a sound-attenuating chest; masking noise was generated by a rotary fan. An Acornmicrocomputer programmed in Arachnid BASIC (CeNeS Ltd, Cambridge, UK), located in an adjoining room, controlled the schedules and recorded the behavioural data.

### 2.3 Behavioural training

The rats were randomly allocated to four groups: ‘adjusting-delay’ (*n* = 12), ‘adjusting-magnitude’ (*n* = 12), ‘no-delay’ (*n* = 10) and ‘deprivation-only’ (*n* = 10). Two weeks before the start of the experiment, the food-deprivation regimen was introduced and the rats were gradually reduced to 80% of their free-feeding body weights. Then the rats in the adjusting-delay, adjusting-magnitude and no-delay groups were trained to press two levers (A and B) for a sucrose reinforcer (50 μl of a 0.6 M solution), and were exposed to a discrete-trials continuous reinforcement schedule in which the two levers were presented in random sequence for three sessions. The positions of levers A and B (left versus right) were counterbalanced across subjects within each group. After this initial training, they underwent daily training sessions under the discrete-trials schedules described below for the remainder of the experiment. In each schedule, experimental sessions comprised 56 trials that were initiated at 30-s intervals; in each case, reinforcer delivery was followed an inter-trial interval in which the chamber remined in darkness until the start of the next trial.

**Fig. 1:**
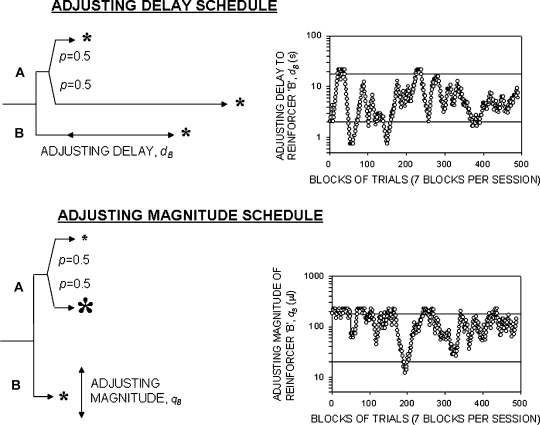
*Left-hand diagrams*: Illustration of the adjusting-delay and adjusting-magnitude schedules. *Adjusting-delay schedule*. In free-choice trials, a response on lever A resulted in the delivery of a reinforcer after a short or a long delay, with equal probability; a response on lever B resulted in the delivery of a reinforcer after a delay *d*_B_, the length of which was adjusted in accordance with the subject's choices (see text for details). *Adjusting-magnitude schedule*. A response on lever A resulted in the immediate delivery of a small or a large reinforcer, with equal probability; a response on lever B resulted in the immediate delivery of a reinforcer whose size *q*_B_ was adjusted in accordance with the subject's choices (see text for details). *Right-hand graphs*: representative performances of individual rats trained under each schedule; *ordinate* adjusting delay (*d*_B_, s) or magnitude (*q*_B_, μl) in successive blocks of trials during 60 training sessions (7 blocks per sessio). Horizontal lines show the two standard delays or magnitudes prescribed for reinforcer A.

*Adjusting-delay schedule* (*n* = 12). Each experimental session consisted of seven blocks of eight trials. Four trials of each block were forced-choice trials in which each lever was presented alone in random sequence. The other four trials were free-choice trials in which both levers were presented. The beginning of each trial was signalled by illumination of the central light above the reinforcer recess. After 2.5 s the lever or levers (depending on the type of trial) were inserted into the chamber. When a lever-press occurred, the lever(s) were withdrawn, the central light was extinguished, and the light located above the lever that had been depressed was illuminated. This light remained illuminated until the delivery of the reinforcer, and was then extinguished. The chamber remained in darkness until the start of the following trial. If no lever-press occurred within 5 s of the lever(s) being inserted, the lever(s) were retracted and the central light extinguished. The adjusting-delay schedule is illustrated in Fig. 1 (upper diagram). A response on lever A initiated a fixed delay, *d*_A_ (2 s or 18 s with equal probability of occurrence [*P* = 0.5 in each case]), following which 50 μl of the 0.6 M sucrose solution was delivered. A response on lever B initiated a variable delay, *d*_B_ (manipulated across blocks of trials during each session: see below), after which 50 μl of the same sucrose solution was delivered. The duration of the variable delay *d*_B_ was determined according to the proportion of responses made on the two levers in the free-choice trials of the previous block. If lever A was chosen in three or four free-choice trials of block *n*, *d*_B_ was reduced by 20% in block *n* + 1; if lever B was chosen in three or four free-choice trials of block *n*, *d*_B_ was increased by 20% in block *n* + 1; if levers A and B were each chosen in two free-choice trials in block *n*, *d*_B_ remained unchanged in block *n* + 1. The value of *d*_B_ in the first block of each session was determined in the same way by the choices made in the final block of the previous session. Maximum and minimum values of *d*_B_ were set at 22 s and 0.75 s.

*Adjusting-magnitude schedule* (*n* = 12). The adjusting-magnitude schedule is illustrated in Fig. 1 (lower diagram). No delays were imposed on either reinforcer (i.e. *d*_*A*_ = *d*_B_ = 0). A response on lever A was associated with the presentation of a reinforcer of fixed magnitude, *q*_A_ (20 μl or 180 μl, with equal probability of occurrence [*P* = 0.5 in each case]), whereas a response on lever B produced a reinforcer of variable magnitude, *q*_B_. The variable magnitude was adjusted in increments or decrements of 20%, in the same manner as was used to adjust *d*_B_ in the adjusting-delay schedule. The maximum and minimum values of *q*_B_ were 220 μl and 8 μl.

*No*-*delay schedule* (*n* = 10). Levers A and B were presented in a series of forced-choice trials in which each lever was presented alone in random sequence. A response on lever A or lever B resulted in the delivery of the reinforcer (50 μl of the 0.6 M sucrose solution) without delay. The number of trials and the duration of the session were the same as in the adjusting-delay and adjusting-magnitude schedules; however, the no-delay schedule did not entail choice between levers nor was there any variation of delay or magnitude of reinforcement.

*Deprivation*-*only* (*n* = 10). Ten rats underwent the same food restriction regimen, but did not undergo any behavioural training.

Experimental sessions were carried out 7 days a week, at the same time each day, during the light phase of the daily cycle (between 08:00 and 13:00 h). Behavioural training continued for 70 sessions.

### 2.4 Immunohistochemistry

Ninety minutes after the final session of the behavioural experiment, the rats were deeply anesthetised with sodium pentobarbitone, and perfused transcardially with 50 ml of 0.1 M phosphate buffered saline (PBS) at pH 7.4, followed by 50 ml of 4% formol PBS. The brains were removed from the skulls and fixed in formol PBS for 4 h, after which they were kept in 30% sucrose in 0.1 M PBS at 4 °C for 48 h. Coronal sections (40 μm) were taken through the brain using a freezing microtome and collected in 0.1 M PBS.

Immunohistochemistry was performed on coronal sections taken through the prefrontal cortex and corpus striatum (see below). The protocol used was an adaptation of the method described by Castro et al. [10]. Freshly sliced sections were rinsed in 0.1 M PBS and placed in 0.3% H_2_O_2_ (Sigma–Aldrich) in PBS for 30 min to inhibit endogenous peroxidase activity. They were then placed in a blocking solution [3% normal goat serum (NGS) (Vector Laboratories, Peterborough, UK) in PBS with 0.3% triton-X (PBS-T)] for 1 h to reduce background staining. Then the sections were incubated for 48 h at 4° C in the primary antibody [polyclonal rabbit anti-Fos (1:5000, Calbiochem, San Diego, CA) in PBS-T with 3% NGS]. After washing in PBS-T, the sections were incubated in biotinylated secondary antibody [goat anti-rabbit (1:600 Sigma, USA)] for 2 h. After further rinsing with PBS, they were incubated for 1 h in avidin–biotin-horseradish peroxidase complex (1:200, ABC-Elite, Vector Laboratories) in PBS. After further rinsing in PBS, they were placed in a chromagen solution (0.05% diaminobenzidine [Sigma–Aldrich] and 0.01% H_2_O_2_ [Sigma–Aldrich]) for approximately 5 min. The reaction was observed visually and stopped by rinsing in PBS. The sections were floated on to chrome-gelatine-coated slides and mounted with DPX.

**Fig. 2:**
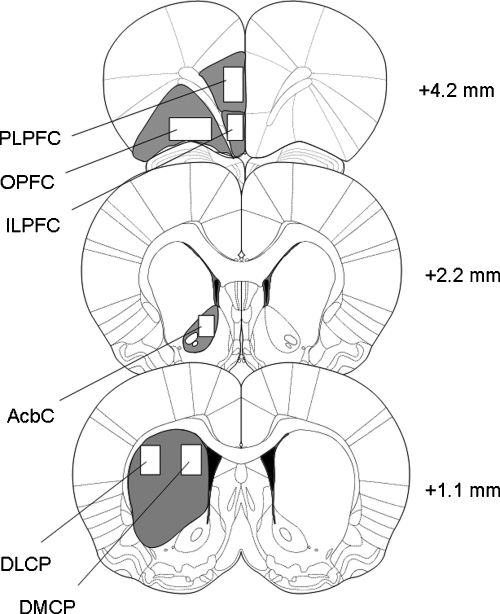
Diagrams of coronal sections of the rat brain showing the areas analysed; the distance anterior to bregma is shown to the right of each section (from Paxinos and Watson [31]). White rectangles indicate the standard areas selected for counting Fos-positive units. PLPFC: prelimbic prefrontal cortex; OPFC: orbital prefrontal cortex; ILPFC: infralimbic prefrontal cortex; AcbC: nucleus accumbens core; DLCP: dorsolateral caudate-putamen; DMCP: dorsomedial caudate-putamen.

Fos-positive cells were identified by the dark reaction product confined to the nucleus. The Fos-positive cells were counted, for a number of areas of interest, with the aid of the ImageJ software (Wayne Rasband, National Institutes of Health, USA). The areas analysed were: infralimbic (ILPFC), prelimbic (PLPFC) and orbital (OPFC) prefrontal cortex, the core of the nucleus accumbens (AcbC) and the dorsomedial (DMCP) and dorsolateral (DMCP) caudate-putamen. The areas analysed are shown in Fig. 2. The reliability of the counting procedure was assessed by four investigators who carried out blind counts of Fos-positive cells in 144 randomly selected and coded slides. The overall inter-rater product-moment correlation coefficient was 0.92.

### 2.5 Data analysis

*Behavioural data*. For each rat in the adjusting-delay group, *d*_B(50)_ was defined as the mean value of *d*_B_ in the last 10 sessions of training. Similarly, for each rat in the adjusting-magnitude group, *q*_B(50)_ was defined as the mean value of *q*_B_ in the last 10 sessions of training. These data are presented below for descriptive purposes only; no between-group comparisons of the behavioural measures are appropriate in this experiment.

*Immunohistochemical data*. Fos-positive cells were counted as described above. The numbers of Fos-positive cells in each area of interest were compared between groups by ANOVA, followed, in the case of a significant *F*-ratio (*P* < 0.05), by multiple comparisons with the control (deprivation-only) group using Dunnett's test. In addition, comparisons were made between the adjusting-delay and adjusting-magnitude groups using the least significant difference test with a conservative criterion of *P* < 0.01 (two-tailed).

## 3 Results

### 3.1 Behavioural data

**Fig. 3:**
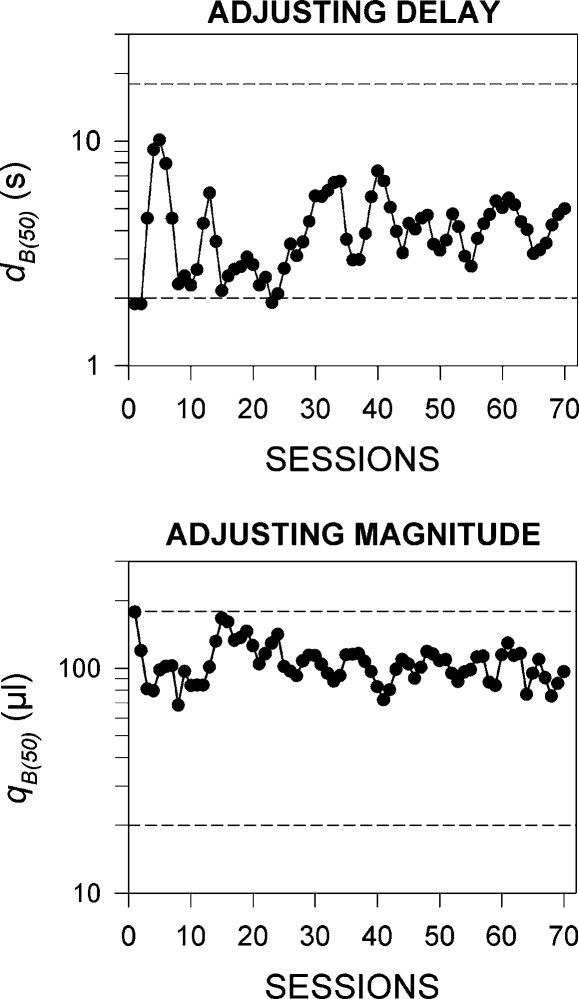
Group mean data from the adjusting-delay and adjusting-magnitude schedules. *Adjusting-delay schedule.* Delays to reinforcer B (*d*_B_) in successive sessions. *Ordinate*: *d*_B_ (s, logarithmic scale); *abscissa*: sessions. The horizontal broken lines show the two fixed values of *d*_A_ (2 s and 18 s). *Adjusting-magnitude schedule.* Sizes of reinforcer B (*q*_B_) in successive sessions. *Ordinate*: *q*_B_ (μl, logarithmic scale); *abscissa*: sessions.

Examples of individual rats’ performance under the adjusting-delay and adjusting-magnitude schedules is shown in Fig. 1. The group mean data are shown in Fig. 3. In the adjusting-delay schedule, the mean value of *d*_B_ (±SEM) in the last 10 session was 4.1 ± 0.9 s. In the adjusting-magnitude schedule, the mean value of *q*_B_ was 112 ± 27 μl. The numbers of reinforcers obtained by the rats in the three groups exposed to operant training were close to the maximum value of 56 reinforcers per session (adjusting-delay group: 54.9 ± 0.8; adjusting-magnitude group: 55.9 ± 0.1; no-delay group: 55.6 ± 0.5).

### 3.2 Immunohistochemical data

**Fig. 4:**
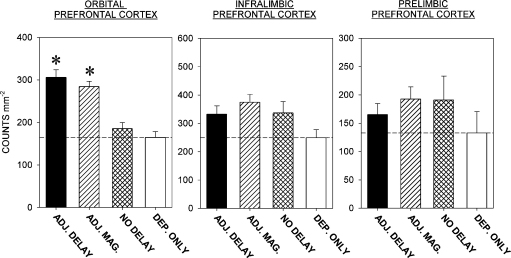
Density of Fos-positive units counted in the three prefrontal cortical regions: *ordinates*: counts mm^−2^. Columns show the group mean data (+SEM) for the rats trained under the adjusting-delay (ADJ. DELAY), adjusting-magnitude (ADJ. MAG.) and no-delay schedules, and the control group of rats exposed to the food-deprivation condition without behavioural training (DEP. ONLY). The horizontal line indicates the group mean data from the control group. The rats trained under the ADJ. DELAY and ADJ. MAG. schedules showed enhanced Fos expression in the orbital prefrontal cortex compared to the control group (*P < 0.05: see text).

**Fig. 5:**
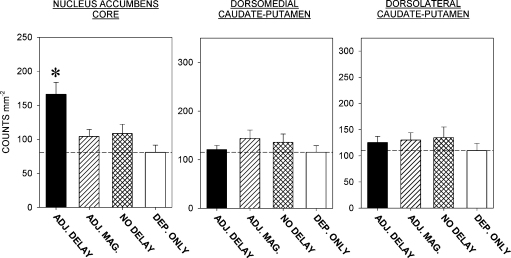
Density of Fos-positive units counted in the three striatal regions (conventions as in Fig. 4). The rats trained under the ADJ. DELAY schedule showed enhanced Fos expression in the nucleus accumbens core compared to the control group (*P < 0.05: see text).

**Fig. 6:**
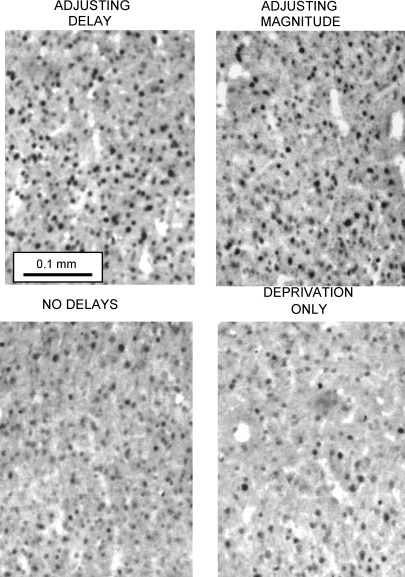
Examples of Fos expression in the orbital prefrontal cortex in rats from the four groups. Note the greater number of Fos-positive units (dark points) in the samples taken from the rats trained under the adjusting-delay and adjusting-magnitude schedules compared to the rat exposed to the ‘deprivation-only’ condition (see text).

**Fig. 7:**
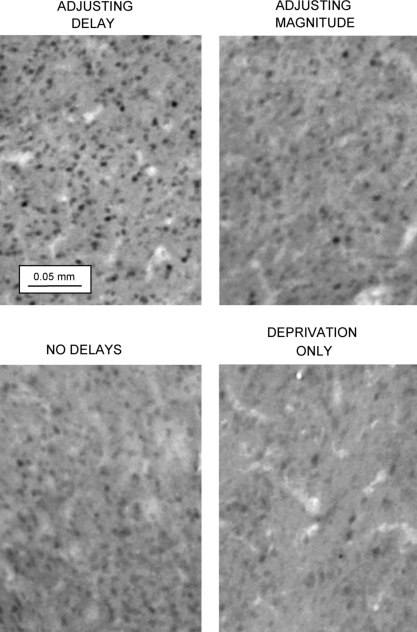
Examples of Fos expression in the nucleus accumbens core in rats from the four groups. Note the greater number of Fos-positive units (dark points) in the samples taken from the rat trained under the adjusting-delay schedule compared to the rat exposed to the ‘deprivation-only’ condition (see text).

The densities of Fos-positive nuclei (counts mm^−2^) in the three cortical and the three striatal regions of interest in the four groups of rats are shown in Figs. 4 and 5, respectively. Representative photomicrographs from the OPFC and AcbC are shown in Figs. 6 and 7.

*OPFC*. There was a significant effect of group [*F*(3,40) = 21.7, *P* < 0.001]. Multiple comparisons with the control (deprivation-only) group indicated that the rats trained under both the adjusting-delay and adjusting-magnitude schedules, but not those trained under the no-delay schedule, showed enhanced Fos expression. There was no significant difference between the Fos expression seen in the rats exposed to the adjusting-delay and adjusting-magnitude schedules.

*ILPFC*. No significant between-group differences in Fos expression were seen in this region [*F*(3,40) = 2.7, *P* > 0.05].

*PLPFC*. No significant between-group differences in Fos expression were seen in this region [*F* < 1].

*AcbC*. There was a significant effect of group [*F*(3,40) = 7.3, *P* < 0.001]. Multiple comparisons with the control (deprivation-only) group indicated that the rats trained under the adjusting-delay schedule, but not those trained under the adjusting-magnitude and no-delay schedules, showed enhanced Fos expression. Fos expression in the rats exposed to the adjusting-delay schedule was significantly greater than that seen in the rats exposed to the adjusting-magnitude schedule.

*DMCP*. No significant between-group differences in Fos expression were seen in this region [*F* < 1].

*DLCP*. No significant between-group differences in Fos expression were seen in this region [*F* < 1].

## 4 Discussion

Fos expression in the OPFC was significantly enhanced in rats exposed to the adjusting-delay and adjusting-magnitude schedules, compared to the levels of Fos expression seen in the rats belonging to the control (deprivation-only) group. This enhancement of Fos expression was presumably not brought about by repetitive reinforcer consumption, nor by repetitive execution of the operant response, since no significant enhancement was seen in rats exposed to the no-delay schedule, which entailed the same number of responses and reinforcer deliveries as the two adjusting schedules. A plausible interpretation of the present finding is that choice behaviour involving comparisons between either different delays to reinforcement or different reinforcer sizes involves substantial activation of the OPFC. Based on this interpretation, the present results are consistent with the results of previous experiments in which lesions of the OPFC were found to disrupt inter-temporal choice behaviour [9,16,17,30,38,43], and, in particular, with the results of an experiment in which quantitative analysis of choice behaviour indicated that destruction of the OPFC had a dual effect on delay discounting and sensitivity to reinforcer size [16]. The involvement of the OPFC in choice behaviour appears to be regionally specific, in the sense that no comparable enhancement of Fos expression was seen in two cortical regions adjacent to the OPFC, the ILPFC and the PLPFC.

Exposure to the adjusting-delay schedule also produced a significant enhancement of Fos expression in the AcbC, compared to the levels seen in the control (deprivation-only) and no-delay groups. Unlike the OPFC, however, there was no detectable enhancement of Fos expression in the AcbC of rats exposed to the adjusting-magnitude schedule. This suggests that the AcbC is involved in choice behaviour based on comparisons of different delays to reinforcement, but not in choice behaviour based on comparisons of different reinforcer sizes. This conclusion is consistent with previous findings on the effects of destruction of the AcbC on inter-temporal choice. Several studies have shown that lesions of the AcbC disrupt inter-temporal choice [1,4,8,11]. Moreover, quantitative analysis of inter-temporal choice suggests that lesions of this structure have a selective effect on the process of delay discounting, and do not alter rats’ sensitivity to reinforcer size [4,5].

There do not appear to have been any previous studies investigating the effect of destruction of the dorsal striatum on inter-temporal choice. The present results do not provide any evidence for an involvement of the dorsal striatum in inter-temporal choice, since the enhancement of Fos expression seen in the AcbC did not extend into the dorsal striatum (DMCP and DLCP). However, a recent functional magnetic resonance neuroimaging study with human subjects found evidence for activation of the dorsal striatum, but not the ventral striatum (AcbC), during performance of an inter-temporal choice task [32]. Whether or not this reflects a consistent difference between the neural regulation of inter-temporal choice in rats and humans is a question that awaits further investigation.

Taken together, the present results are consistent with the notion that the OPFC may have an important role in the integration of information about different features of reinforcing outcomes, including their sizes and delays, which may be of particular importance in guiding inter-temporal choice. The AcbC may play a more restricted role in inter-temporal choice, but may especially important in the process of delay discounting. However, before accepting this conclusion, it is necessary to consider some methodological factors that might have affected the results. It seems safe to assume that differences in the number of reinforcers and the overall rate of reinforcement did not contribute to the difference in Fos expression between the two adjusting schedules, since in both schedules experimental session comprised 56 trials that were initiated at 30-s intervals. The fact that that the adjusting-magnitude schedule did not entail any delays to reinforcement strongly suggests that sensitivity to delay was responsible for the greater Fos expression in the AcbC in the rats exposed to the adjusting-delay schedule, and in view of previous findings based on other inter-temporal choice procedures (see above), it seems likely that the AcbC is engaged in the process of delay discounting, that is the delay-dependent degradation of reinforcer value. However, since no delays were imposed in the adjusting-magnitude schedule, the possibility cannot be excluded that the differences in Fos expression in the AcbC associated with the two adjusting schedules reflected the mere presence of delays, rather than the process of delay discounting. One way of addressing this issue in a future experiment might be to examine the effect of a range of fixed delays to reinforcement in the adjusting-magnitude schedule on Fos expression in the AcbC.

Another feature of the adjusting schedules that deserves mention is the probabilistic nature of the reinforcer delays or magnitudes associated with lever A. In the adjusting-delay schedule, selection of lever A was followed by a 2- or an 18-s delay, each with a probability of 0.5. Similarly, in the adjusting-magnitude schedule, selection of lever A resulted in immediate delivery of a 20- or 180-μl reinforcer, each with a probability of 0.5. Lesions of both the OPFC and the AcbC have been found to alter rats’ sensitivity to probabilistic reinforcement. However, in both cases this has only been demonstrated in the case of reinforcers whose probability of *occurrence* is less than 1.0 (i.e. in cases where there is a greater-than-zero probability of a response not being followed by any reinforcer) [7,17,30]. It remains to be seen whether the OPFC and AcbC contribute to rats’ appraisal of the probabilistic nature of particular features of reinforcers, such as their delays or sizes.

Although there have been numerous reports that lesions of the OPFC can have profound effects on inter-temporal choice behaviour, there is a disconcerting lack of consistency between the types of effect seen in different studies. For example, some studies found that OPFC lesions resulted in diminished ‘tolerance to delay of reinforcement’ [30,38], whereas other studies found the opposite effect [16,17,43]. The present finding suggests a possible explanation for this apparent inconsistency between the results of different experiments. The enhancement of Fos expression induced by both the adjusting-delay and the adjusting-magnitude schedules suggests that the OPFC may be involved in both delay discounting and sensitivity to reinforcer size. Since most conventional inter-temporal choice tasks entail a trade-off between reinforcer size and delay, it may not be surprising that an intervention that disrupts both these processes may give rise to paradoxical effects, depending on whether the effect of the lesion on delay discounting overrides the effect on size sensitivity, or vice versa. Indeed, it has been shown that interventions that simultaneously increase the rate of delay discounting and reduce the organism's sensitivity to reinforcer size tend to bias choice towards the smaller and earlier of two reinforcers when the delay to that reinforcer is relatively short, but tend to have the opposite effect when the delay to that reinforcer is increased [13,29]. Such considerations indicate the need for experimental protocols that allow clear separation of the processes of delay discounting and size sensitivity. Parametric designs based on mathematical models of inter-temporal choice provide one way of addressing this problem [14,28,29]. Such designs are applicable to experiments whose aim is to investigate the effect of neurobiological interventions on inter-temporal choice. However, they are not suitable for experiments such as the present one, whose aim is to identify acute neurochemical or physiological correlates of the two processes; such experiments require experimental protocols that allow exclusive engagement of one process or the other. The adjusting-delay schedule, which did not involve variation of reinforcer size, and the adjusting-magnitude schedule, which did not entail different delays to reinforcement, provided a means of assessing delay discounting and size sensitivity independently of one another, a facility that is precluded by most conventional inter-temporal choice schedules. Although this approach has been used extensively in behaviour analytic studies of inter-temporal choice [19,22,23,25,26], it does not yet have a track record in neurobiological studies. Locey and Dallery [20] recently used the approach to demonstrate that acute systemic treatment with nicotine affected inter-temporal choice by altering size sensitivity rather than directly affecting delay discounting. Their results and the present findings are encouraging for the future use of the method; however, much further work is clearly needed in order to assess its reliability and general applicability to neurobehavioural investigations of inter-temporal choice.

Some comment is needed on the behavioural data. The mean value of the indifference delay, *d*_B(50)_ (4.1 s) was substantially smaller than the arithmetic mean of the two standard delays, *d*_A(SHORT)_ and *d*_A(LONG)_ (10 s). This finding is consistent with the results of many previous studies [19,22,23,26], and is generally regarded as a demonstration of the non-linear (probably hyperbolic [2,3,14,24,27,34]) relation between reinforcer value and delay of reinforcement. In contrast, the mean value of the indifference magnitude, *q*_B(50)_ (112 μl) was close to the arithmetic mean of the two standard magnitudes, *q*_A(SMALL)_ and *q*_(LARGE)_ (100 μl). This result, which suggests linear averaging of reinforcing magnitudes in the computation of overall reinforcer value, was unexpected, in view of recent evidence for a non-linear relation between reinforcer size and reinforcer value [29,36]. The reason for this discrepancy needs further investigation. However, it should be noted that only a single pair of standard reinforcer sizes was used in this experiment; the use of a broader range of magnitudes is no doubt needed in order to assess the nature of the relation between reinforcer size and reinforcer value.

In summary, adjusting-delay and adjusting-magnitude schedules were used in an attempt to isolate the separate influences of delay discounting and size sensitivity on inter-temporal choice. Exposure to the adjusting-delay schedule was associated with enhanced Fos expression in the OPFC and AcbC, whereas exposure to the adjusting-magnitude schedule was associated with enhanced Fos expression in the OPFC but not the AcbC. The results are consistent with the notion that the AcbC is involved in delay discounting, whereas the OPFC may contribute to the computation of reinforcer value based on multiple features of reinforcing stimuli, including both the sizes and the delays of reinforcers [37,41].
